
JOURNAL INFORMATION
==============================
NLM Title Abbreviation: Hum Brain Mapp
Journal ID: Hum Brain Mapp
NLM Journal ID: 9419065
Journal ID: HBM

Human Brain Mapping

ISSN: 1065-9471
EISSN: 1097-0193
Publisher: Wiley

ARTICLE INFORMATION
==============================
PMCID: PMC11474575
DOI: 10.1002/hbm.25938
Article ID: HBM25938
Article version: 1
Subjects: Issue Information, Issue Information

Editorial board ‐ TOC

Electronic publication date: 2023 Oct 2
Publication date: 2023 Nov
Volume: 44
Issue: 16
First page: 5199
Last page: 5201
Copyright: © 2023 The Authors. Human Brain Mapping published by Wiley Periodicals LLC.
License: This is an open access article under the terms of the http://creativecommons.org/licenses/by/4.0/ License, which permits use, distribution and reproduction in any medium, provided the original work is properly cited.
License URL: https://creativecommons.org/licenses/by/4.0/

Figure count: 0
Table count: 0
Page count: 7

source-schema-version-number: 2.0
cover-date: November 2023
details-of-publishers-convertor: Converter:WILEY_ML3GV2_TO_JATSPMC version:6.4.9 mode:remove_FC converted:15.10.2024

==============================

### HUMAN BRAIN MAPPING

Open Access

Volume 44, Issue 16, November, 2023

View this journal online at wileyonlinelibrary.com

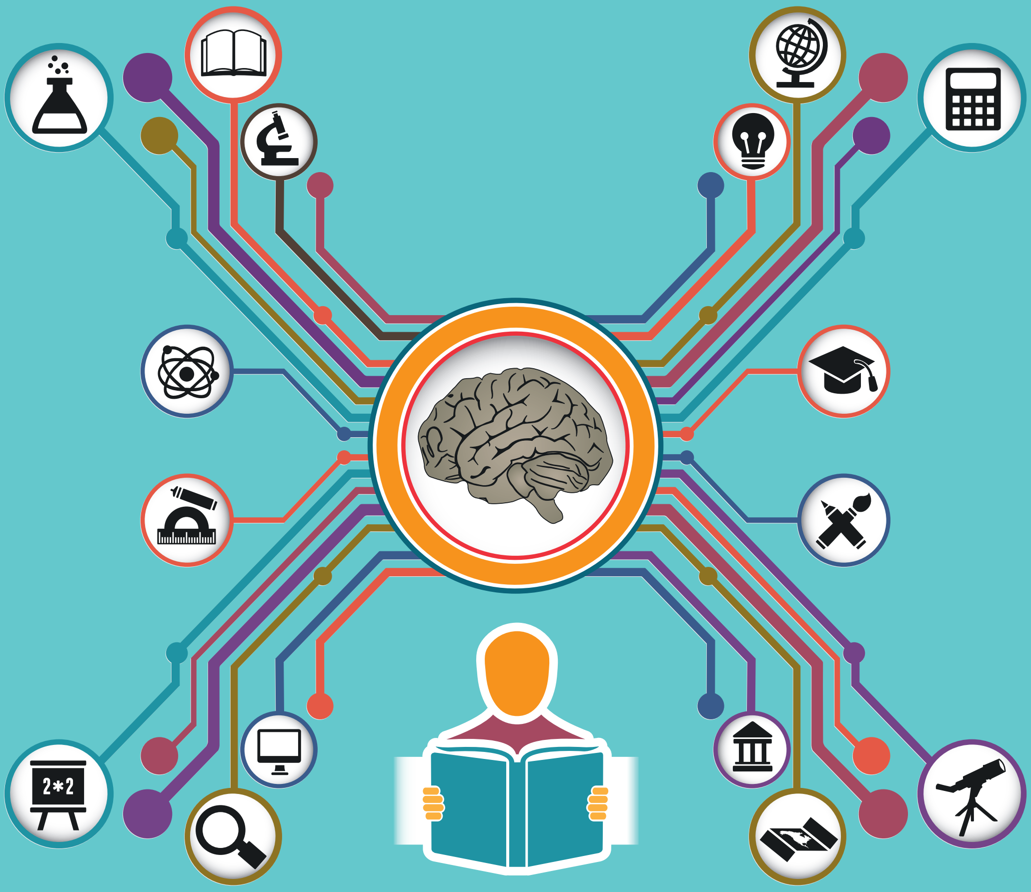

WILEY

ISSN 1097-0193

### HUMAN BRAIN MAPPING

### A Journal Devoted to Functional Neuroanatomy and Neuroimaging

### *Editor-in-Chief*

**Simon B. Eickhoff**
Institute for Systems Neuroscience
Heinrich-Heine University, Düsseldorf, Germany

### *Deputy Editors-in-Chief*

**Gary Egan**
Monash Biomedical Imaging
Monash University, Victoria, Australia

**Sophia Frangou**
Icahn School of Medicine
at Mount Sinai, New York, USA

**Martijn van den Heuvel**
Brain Center Rudolf Magnus,
University of Medical Center,
Utrecht, Netherlands

### *Associate Editors*

**Annabel Chen**
Nanyang Technological University, Singapore

**Michael J. Farrell**
Monash University, Victoria, Australia

**Massimo Filippi**
Università Vita-Salute San Raffaele, Milan,
Italy

**Sophia Frangou**
Icahn School of Medicine at Mount Sinai
New York, USA

**Cheryl L. Grady**
Rotman Research Institute
Toronto, Canada

**Joachim Gross**
Centre for Cognitive Neuroimaging
University of Glasgow, Glasgow, UK

**Martin A. Lindquist**
John Hopkins, Bloomberg School of Public
Health, MD, USA

**Tomáš Paus**
Rotman Research Institute
Toronto, Canada

**Indira Tendolkar**
Radboud University, Nijmegen,
The Netherlands

**Paul Thompson**
Laboratory of Neuro Imaging,
University of Southern California,
Los Angeles, CA, USA

**Helen Zhou**
Yong Loo Lin School of Medicine, National
University of Singapore, Singapore

### *Editorial Board*

**Susan Bookheimer**
Center for Cognitive Neuroscience
UCLA School of Medicine, USA

**Allen R. Braun**
NIDCD, National Institutes of Health
Bethesda, USA

**Christian Buechel**
Univ-Krankenhaus Eppendorf
Hamburg, Germany

**Marco Catani**
King's College London, UK

**Franco Cauda**
University of Turin, Italy

**Vincent Clark**
University of New Mexico, USA

**Olivier David**
Université Joseph Fourier, France

**Mark D'Esposito**
University of California, Berkeley,
USA

**Julien Doyon**
Functional Neuroimaging Unit
University of Montreal, Canada

**Eugene Duff**
University of Oxford, UK

**Timothy Q. Duong**
Research Imaging Institute,
University of Texas Health Science
Center, USA

**Paul Fletcher**
University of Cambridge, UK

**Jia-Hong Gao**
Brain Research Imaging
Center The University of Chicago,
USA

**David C. Glahn**
Yale University School of Medicine
Hartford, USA

**Gregory S. Hickok**
Center for Cognitive
Neuroscience University of California,
Irvine, USA

**Barry Horwitz**
NIDCD, National Institutes of Health
USA

**Peter Jezzard**
John Radcliffe Hospital
University of Oxford, UK

**David N. Kennedy**
Harvard Medical School, USA

**Neda Ladbon-Bernasconi**
Montreal Neurological Institute
Canada

**Nicholas Lange**
Harvard Medical School, USA

**Tatia M. C. Lee**
State Key Laboratory of Brain &
Cognitive Sciences, The University of
Hong Kong

**Louis Lemieux**
UCL Institute of Neurology, UK

**Bernard Mazoyer**
CNRS/CEA, Université de Bordeaux
France

**Gregory McCarthy**
Yale University, USA

**A. Randy McIntosh**
Rotman Research Institute
Toronto, Canada

**Janine Mendola**
McGill Vision Research
McGill University, Canada

**Thomas Nichols**
University of Warwick, UK

**William Penny**
Wellcome Trust Centre for
Neuroimaging, UCL Institute of
Neurology, UK

**Laurent Petit**
CNRS, Université de Bordeaux,
France

**Russel A. Poldrack**
Stanford Unversity, USA

**Jean-Baptiste Poline**
Imagen Orsay Service Hospitalier
Frédéric Joliot, France

**Stephen Smith**
Center for Functional MRI of the
Brain, University of Oxford, UK

**Elizabeth Sowell**
Children'sHospital Los Angeles, USA

**Li Hai Tan**
Shenzhen Institute of Neuroscience
Shenzhen, P.R. China

**Kate Watkins**
John Radcliffe Hospital
University of Oxford, UK

**Richard G. Wise**
Cardiff University, UK

**Roger Woods**
UCLA School of Medicine, USA

**Mark Woolrich**
Oxford Centre for Human Brain
Activity, University of Oxford, UK

**Jinhu Xiong**
University of Iowa, USA

**Robert Zatorre**
MontrealNeurological Institute, Canada

**Open Access and Copyright**
All articles published by *Human Brain Mapping* submitted after January 1, 2020 are fully open access: immediately freely available to read, download, and share. All articles accepted from August 6, 2019 are published under the terms of a Creative Commons license.
Copyright on any research article published by *Human Brain Mapping* is retained by the author(s).

Further information about open access licenses and copyright can be found at: https://authorservices.wiley.com/open-research/open-access/index.html. Information on the licenses used by *Human Brain Mapping* can be found at: https://onlinelibrary.wiley.com/journal/10970193.

**Disclaimer**
The Publisher and Editors cannot be held responsible for errors or any consequences arising from the use of information contained in this journal; the views and opinions expressed do not necessarily reflect those of the Publisher and Editors, neither does the publication of advertisements constitute any endorsement by the Publisher and Editors of the products advertised. Wiley Open Access articles posted to repositories or websites are without warranty from Wiley of any kind, either express or implied, including, but not limited to, warranties of merchantability, fitness for a particular purpose, or non-infringement. To the fullest extent permitted by law Wiley disclaims all liability for any loss or damage arising out of, or in connection, with the use of or inability to use the content.

HUMAN BRAIN MAPPING

A Journal Devoted to Functional Neuroanatomy and Neuroimaging

VOLUME 44, NUMBER 16, NOVEMBER 2023

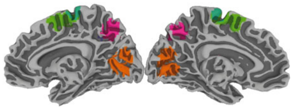

*See article by, Chen and Maitra, pages 5309-5335, this issue*

### CONTENTS

◆ *5202* An automated pipeline for obtaining labeled ICA-templates corresponding to functional brain systems
**Marlene Tahedl and Jens V. Schwarzbach**
***Published online 30 July 2023***

◆ *5212* The need for hemispheric separation in pairwise structural disconnection studies
**Lisa Röhrig, Hannah Rosenzopf, Sofia Wöhrstein, and Hans-Otto Karnath**
***Published online 4 August 2023***

◆ *5221* Retinotopic connectivity maps of human visual cortex with unconstrained eye movements
**Gene Tangtartharakul, Catherine A. Morgan, Simon K. Rushton, and D. Samuel Schwarzkopf**
***Published online 9 August 2023***

◆ *5238* Genetic and environmental influence on resting state networks in young male and female adults: a cartographer mapping study
**Arman P. Kulkarni, Gyujoon Hwang, Cole J. Cook, Rosaleena Mohanty, Akhil Guliani, Veena A. Nair, Barbara B. Bendlin, Elizabeth Meyerand, and Vivek Prabhakaran**
***Published online 20 December 2022***

◆ *5294* High-accuracy machine learning techniques for functional connectome fingerprinting and cognitive state decoding
**Andrew Hannum, Mario A. Lopez, Saúl A. Blanco, and Richard F. Betzel**
***Published online 27 July 2023***

***(continued on overleaf)***

(*continued from overleaf*)

◆ *5309* A practical model-based segmentation approach for improved activation detection in single-subject functional magnetic resonance imaging studies
**Wei-Chen Chen and Ranjan Maitra**
***Published online 4 August 2023***

◆ *5336* Disability and persistent motor deficits are linked to structural crossed cerebellar diaschisis in chronic stroke
**Stephanie Guder, Fatemeh Sadeghi, Simone Zittel, Fanny Quandt, Chi-un Choe, Marlene Bönstrup, Bastian Cheng, Götz Thomalla, Christian Gerloff, and Robert Schulz**
***Published online 20 July 2023***

◆ *5346* Altered dynamic brain activity and functional connectivity in thyroid-associated ophthalmopathy
**Wen-Hao Jiang, Jun Liu, Jiang Zhou, Qian Wu, Xiong-Ying Pu, Huan-Huan Chen, Xiao-Quan Xu, Fei-Yun Wu, and Hao Hu**
***Published online 29 July 2023***

◆ *5357* The role of neurotransmitters in mediating the relationship between brain alterations and depressive symptoms in patients with inflammatory bowel disease
**Jun Wang, Guangyao Liu, Kun Xu, Kai Ai, Wenjing Huang, and Jing Zhang**
***Published online 2 August 2023***

◆ *5372* Preterm-birth alters the development of nodal clustering and neural connection pattern in brain structural network at term-equivalent age
**Weihao Zheng, Xiaomin Wang, Tingting Liu, Bin Hu, and Dan Wu**
***Published online 4 August 2023***

◆ *5387* Association between gene expression and functional-metabolic architecture in Parkinson's disease
**Zhenxiang Zang, Xiaolong Zhang, Tianbin Song, Jiping Li, Binbin Nie, Shanshan Mei, Zhi'an Hu, Yuqing Zhang, and Jie Lu**
***Published online 22 August 2023***

◆ *5402* Social perception in deaf individuals: A meta-analysis of neuroimaging studies
**Maria Arioli, Cecilia Segatta, Costanza Papagno, Marco Tettamanti, and Zaira Cattaneo**
***Published online 23 August 2023***

◆ *5416* Clinical-functional brain connectivity signature predicts longitudinal symptom improvement after acupuncture treatment in patients with functional dyspepsia
**Tao Yin, Yuzhu Qu, Yangke Mao, Pan Zhang, Peihong Ma, Zhaoxuan He, Ruirui Sun, Jin Lu, Yuan Chen, Shuai Yin, Qiyong Gong, Yong Tang, Fanrong Liang, and Fang Zeng**
***Published online 16 August 2023***

(*continued on overleaf*)

(*continued from overleaf*)

◆ *5429* Single-subject cortical morphological brain networks across the adult lifespan
**Jingxuan Ruan, Ningkai Wang, Junle Li, Jing Wang, Qihong Zou, Yating Lv, Han Zhang, and Jinhui Wang**
***Published online 14 August 2023***

◆ *5450* Abnormal individualized peak functional connectivity toward potential repetitive transcranial magnetic stimulation treatment of autism spectrum disorder
**Jing Jin, Xiu-Qin Wang, Xue Yang, Na Zhao, Zi-Jian Feng, Yu-Feng Zang, and Li-Xia Yuan**
***Published online 11 September 2023***

◆ *5460* Cognitive inflexibility is linked to abnormal frontoparietal-related activation and connectivity in obsessive-compulsive disorder
**Qian Liu, Feng Gao, Xiang Wang, Jie Xia, Gangxuan Yuan, Shuxin Zheng, Mingtian Zhong, and Xiongzhao Zhu**
***Published online 8 September 2023***

◆ *5471* A selection and targeting framework of cortical locations for line-scanning fMRI
**Jurjen Heij, Luisa Raimondo, Jeroen C. W. Siero, Serge O. Dumoulin, Wietske van der Zwaag, and Tomas Knapen**
***Published online 22 August 2023***

◆ *5485* Mapping the macrostructure and microstructure of the in vivo human hippocampus using diffusion MRI
**Bradley G. Karat, Jordan DeKraker, Uzair Hussain, Stefan Köhler, and Ali R. Khan**
***Published online 24 August 2023***

◆ *5504* Adolescents with ADHD and co-occurring motor difficulties show a distinct pattern of maturation within the corticospinal tract from those without: A longitudinal fixel-based study
**Christian Hyde, Ian Fuelscher, Daryl Efron, Vicki A. Anderson, and Tim J. Silk**
***Published online 22 August 2023***

◆ *5514* Erratum to: The neural correlates of value representation: From single items to bundles
***Published online 4 September 2023***
